# Global burden and prediction study of schizophrenia 1990–2030: comparison with China

**DOI:** 10.1186/s12888-025-07168-6

**Published:** 2025-10-09

**Authors:** Zhou Yuan, Chen Bai, Yue li, Jin Zhang, Ping Yu, Fei Jiang

**Affiliations:** 1 Department of General Surgery, Hubei Provincial Clinical Research Center for Umbilical Cord Blood Hematopoietic Stem Cells, Taihe Hospital, Hubei University of Medicine, Shiyan, Hubei 442000 China; 2 Department of Pharmacy , Taihe Hospital, Hubei University of Medicine, Shiyan, China; 3 Office of Administration and Management, Taihe Hospital, Hubei University of Medicine, Shiyan, Hubei 442000 China; 4 Department of Respiratory, Taihe Hospital of Shiyan, Hubei University of Medicine, Renmin Road No. 30, Shiyan, Hubei 442000 China; 5 Department of Information Resources,, Taihe Hospital, Hubei University of Medicine, 442000, Shiyan, China

**Keywords:** Schizophrenia, Global Burden of Disease, Prevalence, Incidence, Disability-adjusted life years

## Abstract

**Objective:**

This study aims to assess and predict global and Chinese schizophrenia burden trends from 1990 to 2030.

**Methods:**

Retrieve data from the 2021 Global Burden of Disease Study to estimate the incidence, prevalence, and disability-adjusted life years (DALYs) of schizophrenia from 1990 to 2021. Establish a joint point analysis and Bayesian age-period-cohort (BAPC) model to predict the burden of schizophrenia in 2030.

**Result:**

In 2021, the global prevalence of schizophrenia was approximately 13.60 million cases, with an age-standardized rate of 275.78 per 100,000 population. The global incidence was around 1.2 0million cases, with a rate of 15.43 per 100,000, and the global DALYs totaled 14.80 million, with a rate of 177.75 per 100,000. In China, the prevalence was about 5.30 million cases (300.81 per 100,000), the incidence was 236,175 cases (18.36 per 100,000), and DALYs were 3.40 million (203.88 per 100,000). From 1990 to 2021, China’s age-standardized prevalence and DALY rates increased, with estimated annual percentage change (EAPCs) of 0.12 and 0.04. However, the global age-standardized incidence rate also decreased, with an EAPC of -0.04. Using the BAPC model, forecasts indicate a rising trend in both global and Chinese schizophrenia prevalence, incidence, and DALYs from 2020 to 2030. By 2030, the global age-standardized prevalence, incidence, and DALYs are projected to reach 280.36, 15.59, and 177.31 per 100,000, respectively. In China, these rates are expected to reach 332.58, 19.87, and 216.67 per 100,000.

**Conclusion:**

The global burden of schizophrenia is increasing, particularly in China, where the trend is especially pronounced. In response to this trend, Mental health education and early identification measures should be promoted for high-burden populations, especially young adults aged 20–34, while mental health support services for men should be strengthened and more gender-sensitive treatment and rehabilitation pathways should be designed. As future burden projections continue to rise, policymakers should optimize resource allocation, accordingly, incorporate research data into mental health planning, and improve accessibility and equity of services for key populations, in order to promote more targeted intervention strategies and effectively alleviate the social and family burden of schizophrenia.

**Supplementary Information:**

The online version contains supplementary material available at https://doi.org/10.1186/s12888-025-07168-6.

## Introduction

Schizophrenia is a severe mental disorder that profoundly affects patients''lives and poses a significant challenge to global public health [1, 2]. Understanding the global burden of schizophrenia and its future trends is crucial for policymakers, healthcare providers, and researchers in effectively allocating resources and developing targeted interventions [3].

Firstly, the global burden of schizophrenia is substantial. Approximately 20 million people worldwide suffer from schizophrenia, with prevalence and incidence rates varying by region [4]. Although the global prevalence of schizophrenia has remained relatively stable over the past few decades, the absolute number of cases has increased due to population growth and aging [5]. Schizophrenia typically manifests in late adolescence or early adulthood, with males experiencing earlier onset and more severe symptoms than females. Schizophrenia is not only a leading cause of disability but also significantly increases Years Lived with Disability (YLDs) [6]. Individuals with schizophrenia have a significantly shortened lifespan, typically dying 10–20 years earlier than the general population, mainly due to comorbid physical health problems and a higher suicide rate [7, 8].

The economic burden of schizophrenia is also immense, encompassing direct medical costs, indirect costs (such as productivity loss), and social care expenses [9]. As prevalence and healthcare demands increase, these costs are expected to rise significantly in the future. Investment and resource allocation in mental health services vary globally, with low- and middle-income countries particularly facing inadequate mental health services, exacerbating the economic burden on patients. Predicting the future burden of schizophrenia requires analyzing historical data and considering demographic changes [10, 11]. By 2030, the number of people with schizophrenia is expected to increase, primarily due to population growth and aging, especially in low- and middle-income countries [12, 13]. There is a need for enhanced attention and resource allocation to mental health globally to meet the increasing demand.

China, as a populous nation, faces a significant burden of schizophrenia. The prevalence of schizophrenia in China is estimated to be like the global average, but due to its large population base, the absolute number of cases is high [14]. In recent years, the Chinese government has made numerous efforts to improve mental health awareness and service levels, yet challenges such as a shortage of professionals, uneven resource distribution, and the stigma associated with mental illness remain, affecting patients''access to care and recovery [15, 16]. While China’s healthcare system has made progress in improving mental health services, challenges persist. The shortage of mental health professionals, unequal distribution of resources, and the stigma surrounding mental illness hinder patients from receiving timely and appropriate treatment [17]. Recently, the Chinese government has introduced policies aimed at strengthening mental health services, including increasing training for mental health professionals and enhancing the accessibility of community mental health services.

The situation of disability and mortality among schizophrenia patients in China is like global trends. However, cultural factors and differences in healthcare infrastructure may influence the management and treatment outcomes of schizophrenia [18]. Traditional cultural values and social stigma often lead to patients and their families being reluctant to seek help openly, further exacerbating the condition and the burden on families. The economic impact of schizophrenia in China is significant, with high medical costs and productivity losses. In recent years, as national attention to mental health has increased, policy reforms and investments in mental health services have aimed at reducing the economic burden of schizophrenia. These measures include strengthening early intervention, providing financial support, and improving the social security system for patients [19].

By 2030, the burden of schizophrenia in China is expected to increase, reflecting global trends. Population aging and growing healthcare demands will require more comprehensive mental health services and strategic planning to mitigate the impact of schizophrenia. China needs to continue strengthening policy implementation, increasing resource investment, and improving the coverage and quality of mental health services to address future challenges [20].

To address the increasing burden of schizophrenia, both globally and in China, it is essential to enhance mental health services and improve their accessibility and quality, especially in underserved areas. Raising mental health awareness and reducing social stigma can encourage patients to seek help actively. Increasing investment in schizophrenia research to develop effective treatments and prevention strategies is crucial. Implementing and strengthening policies that support mental health and protect the rights of schizophrenia patients is also necessary. Through these measures, the global and Chinese healthcare systems can better manage the growing burden of schizophrenia and improve patients''quality of life.

## Methods

### Data sources

The estimates are based on the Global Burden of Disease (GBD) 2021 analytic framework, led by the Institute for Health Metrics and Evaluation (IHME) at the University of Washington, which aims to provide systematic, standardized, and comparable prevalence, incidence, and disability-adjusted life-years (DALYs) for 371 diseases and injuries worldwide. The GBD has a wide range of data sources, including censuses, disease surveillance, outpatient and inpatient data, scientific literature, and mental health epidemiologic surveys, to ensure the comprehensiveness and diversity of information. To handle these heterogeneous data, GBD uses the DisMod-MR 2.1 modeling tool, a multilayer mixed-effects model based on Bayesian inference, which can establish logical consistency between different data indicators so that key indicators such as incidence and prevalence can still be estimated in the absence of data. In the composition of DALYs, considering that schizophrenia does not usually lead directly to death, GBD does not calculate years of life lost (YLL) due to premature death for it, so DALYs are equivalent to quality of life lost due to disability (YLDs), which is estimated based on the number of people with the disease and the distribution of disease severity, which is obtained through the Global Survey of Disability Weights (GSDW). To eliminate the effects of population age structure between countries and time periods, this study uses the GBD World Standard Population for age standardization and reports 95% uncertainty intervals to express the uncertainty in model estimates. This standardized modeling framework provided reliable and internationally comparable burden of disease estimates for this study [21, 22]. This includes annual age-specific data on the prevalence, incidence, mortality, and DALYs of schizophrenia for China and the world during the 1990–2019 period, encompassing absolute numbers, crude rates, and age-standardized rates (ASRs) with 95% confidence intervals (CI) generate the posterior distribution of the estimated parameters by Markov chain Monte Carlo (MCMC) sampling or approximate inference methods. Confidence intervals, the range between the 2.5th percentile and the 97.5th percentile, are extracted from this posterior distribution. If the confidence intervals overlap between different countries, genders, or age groups, this indicates that the differences between these groups may not be statistically significant; conversely, if the confidence intervals do not overlap, this indicates that the differences are more likely to be real. This way of quantifying uncertainty not only reflects the volatility of the data itself but also covers parameter uncertainty in the estimation process of the model and is therefore an important indicator for evaluating the robustness and reliability of the results of the study. Additionally, it includes population data for China and the world across different age groups from 1990 to 2021, as well as projected population data from 2020 to 2030.

### Basic indicators

To measure the disease burden caused by schizophrenia, we collected data on the number of cases, incidence rates, number of prevalent cases, and prevalence rates of schizophrenia globally and in China, across different years, genders, and age groups. We used DALYs and their rates as indicators. Since there is currently no evidence that schizophrenia directly causes patient death, the database on schizophrenia disease burden indicators does not include mortality rates or calculate Years of Life Lost (YLL) due to premature death. We used the GBD world standard population as a reference to estimate the ASR [23].

### Joinpoint regression analysis

We utilized a joinpoint regression model to assess the temporal trends of schizophrenia burden from 1990 to 2021 by calculating the Average Annual Percent Change (AAPC). These models segment the data into multiple intervals and fit linear regression within each segment, effectively capturing changes in trends. The core principle involves identifying the best segmentations to optimally match the observed trends, facilitating the analysis of pattern changes. We employed Joinpoint version 4.9.1 (National Cancer Institute, Rockville, MD, USA) to fit the simplest joinpoint model allowed by the data (i.e., the model with the fewest joinpoints) and calculated the AAPC using the best model [24]. The regression line equation can be represented as y = α + βx + ϵy = α + βx + ϵ, where y = ln (ASR) and x represents the years. The AAPC is computed as 100 × (exp(β) − 1)100 × (exp(β) − 1), where values > 0 indicate an increasing trend and values < 0 indicate a decreasing trend. We considered a *p*-value < 0.05 to be statistically significant [25]. To assess the trend of age-standardized rate (ASR) over time, we used Joinpoint regression analysis. Using a logarithmic link function, the model assumes that the ASR trends exponentially over time, i.e., at a constant Annual Percent Change (APC). This assumption has a strong basis in epidemiologic research and is particularly applicable to a wide range of chronic diseases or health outcomes. The possibility of exponential changes in ASR is due to a combination of factors such as the aging of the population, continued exposure to risk factors, increased screening rates for disease, and advances in treatment. The logarithmically transformed APC can be visualized as a “relative rate of change per year,” which can be easily interpreted and compared in public health surveillance and interventions.

### Construction of Bayesian age-period-cohort model

The Bayesian age-period-cohort (BAPC) model is a powerful statistical tool that aids researchers in understanding trends, particularly changes in disease rates. This model focuses on three key aspects: age (the individual’s current age), period (external factors affecting all individuals), and cohort (individuals born within the same timeframe). Traditional models often struggle to disentangle these intertwined factors, but the BAPC model leverages Bayesian methods to clarify their effects.

By combining prior beliefs with observed data, the BAPC model elucidates how age, period, and cohort influence outcomes while expressing uncertainty in these estimates [26, 27]. Mathematically, the model can be represented as:

$$\begin{aligned} \mathrm y\left(\mathrm a,\mathrm p,\mathrm c\right)\;&=\alpha\left(\mathrm a\right)+\beta\left(\mathrm p\right)+\gamma\left(\mathrm c\right)+\varepsilon\left(\mathrm a,\mathrm p,\mathrm c\right)\gamma\left(\mathrm a,\mathrm p,\mathrm c\right)\\&=\alpha\left(\mathrm a\right)+\beta\left(\mathrm p\right)+\gamma\left(\mathrm c\right)+\varepsilon\left(\mathrm a,\mathrm p,\mathrm c\right) \end{aligned}$$

where y(a,p,c)y(a,p,c) is the observed outcome for a specific combination of age (aa), time period (pp), and birth cohort (cc); α(a)α(a), β(p)β(p), and γ(c)γ(c) denote the age, period, and cohort effects, respectively; and ϵ(a,p,c)ϵ(a,p,c)represents the residual error term [28].

To estimate the effects, the BAPC model employs Bayesian inference, incorporating prior knowledge and updating it with observed data to obtain posterior distributions. These distributions are typically explored through Markov Chain Monte Carlo (MCMC) sampling techniques. The optimal model is selected based on the Deviance Information Criterion (DIC) [29].

Using age-specific population data from 1990 to 2021, projected population data from 2022 to 2030, and the GBD world population age standard, we applied the BAPC model to predict the burden of schizophrenia from 2022 to 2030. Our predictions of the schizophrenia burden were based on past trends, without considering potential changes in risk factors or interventions. The BAPC models were constructed using the''INLA''and''BAPC''packages in R, version 4.4.1 (R Core Team, Vienna, Austria) [30, 31].

## Result

### Changes in the Global Burden of Schizophrenia from 1990 to 2021

**Fig. 1:**
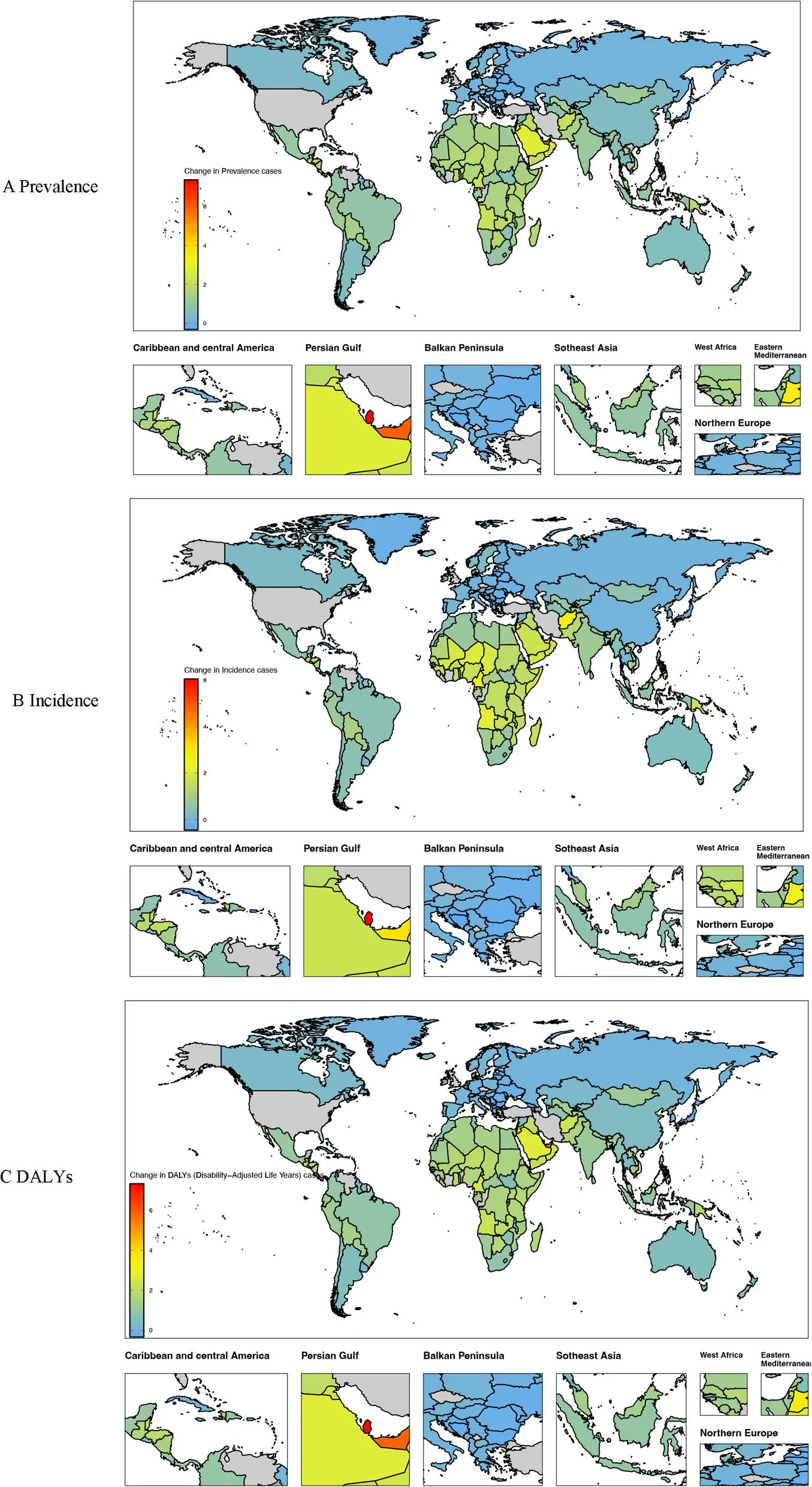
World map of the burden of schizophrenia

Based on (Fig. 1 and Supplementary Material Table S1), it can be concluded that Qatar exhibits the largest relative changes in prevalence, incidence, and DALYs, with values of 7.36, 6.03, and 7.32, respectively. In 2021, the global prevalence of schizophrenia was approximately 13,621,402 cases, with an age-standardized prevalence rate of 275.78 per 100,000 population. The global incidence of schizophrenia was approximately 1,223,221 cases, with an age-standardized incidence rate of 15.43 per 100,000 population. The global schizophrenia DALYs (Disability-Adjusted Life Years) was approximately 14,816,611 cases, with an age-standardized DALY rate of 177.75 per 100,000 population. From 1990 to 2021, the global age-standardized prevalence rate increased with an EAPC (Estimated Annual Percentage Change) of 0.02, the global age-standardized DALYs rate increased with an EAPC of 0.02, while the global age-standardized incidence rate decreased with an EAPC of −0.04 (Table 1).

**Table 1:** Prevalence, incidence and DALYs (Disability-Adjusted Life Years) of schizophrenia in 1990 and 2021, and their annual percentage change 1990–2021

	Prevalence					Incidence		
	Number of cases, 1990	Age-standardized rate per 100,000 population, 1990	Number of cases, 2021	Age-standardized rate per 100,000 population, 2021	Estimated annual percentage change, 1990–2021	Number of cases, 1990	Age-standardized rate per 100,000 population, 1990	Number of cases, 2021
China	3,558,619 (3,076,267,4,080,968)	300.81 (260.98,343.19)	5,322,430 (4,637,003,6,043,640)	312.36 (271.69,356.39)	0.12(0.11,0.15)	251,820 (215,196,291,261)	18.19 (15.71,20.97)	236,175 (201,684,275,708)
Global	13,621,402 (11,333,557,16,166,722)	275.78 (229.94,324.02)	23,182,109 (19,203,759,27,423,876)	277.71 (229.77,329.06)	0.02(0.01,0.03)	883,493 (721,489,1,065,740)	15.64 (13.04,18.62)	1,223,221 (1,008,219,1,473,083)

	Incidence		DALYs				
	Age-standardized rate per 100,000 population, 2021	Estimated annual percentage change, 1990–2021	Number of cases, 1990	Age-standardized rate per 100,000 population, 1990	Number of cases, 2021	Age-standardized rate per 100,000 population, 2021	Estimated annual percentage change, 1990–2021
China	18.36 (15.86,21.18)	0.04(0.02,0.06)	2,329,187 (1,763,156,2,915,190)	195.67 (147.78,244.07)	3,445,845 (2,572,495,4,306,768)	203.88 (152.53,255.67)	0.14(0.11,0.17)
Global	15.43 (12.74,18.62)	−0.04(−0.05, −0.03)	8,762,312 (6,477,261,11,263,750)	176.61 (130.85,226.02)	14,816,611 (10,926,460,19,095,362)	177.75 (131.51,228.8)	0.02(0.01,0.03)

In 2021, the China prevalence of schizophrenia was approximately 5,322,430 cases, with an age-standardized prevalence rate of 300.81 per 100,000 population. The China incidence of schizophrenia was approximately 236,175 cases, with an age-standardized incidence rate of 18.36 per 100,000 population. The China schizophrenia DALYs was approximately 3,445,845 cases, with an age-standardized DALY rate of 203.88 per 100,000 population. From 1990 to 2021, the age-standardized prevalence rate in China increased, with an EAPC of 0.12; the age-standardized DALY rate in China increased, with an EAPC of 0.14; the China age-standardized incidence rate increased, with an EAPC of 0.04, all of which were higher than the global levels (Table 1).

### The burden of schizophrenia in 2021 across different age groups and gender

**Fig. 2:**
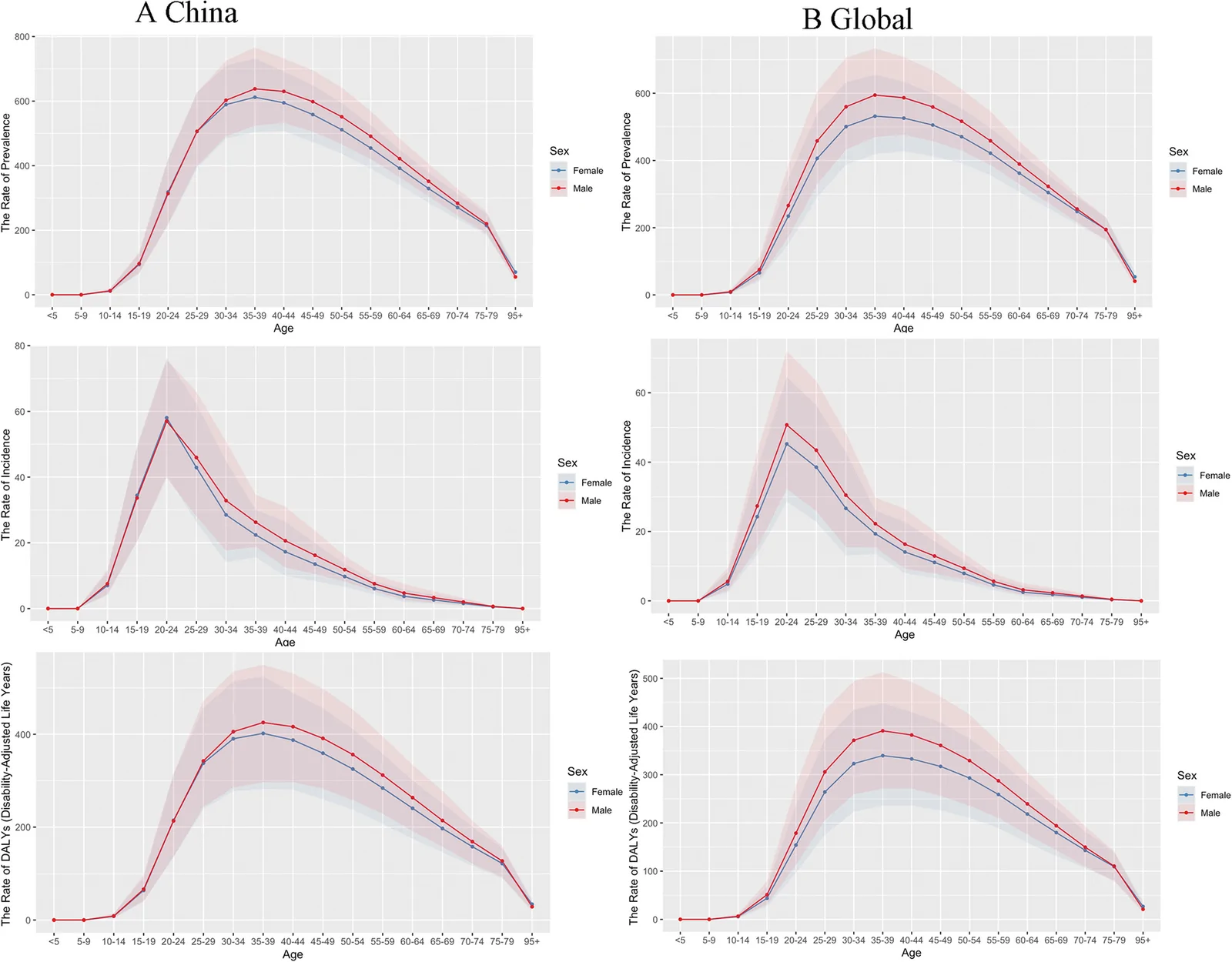
Line plots of the burden of schizophrenia by age and gender

**Fig. 3:**
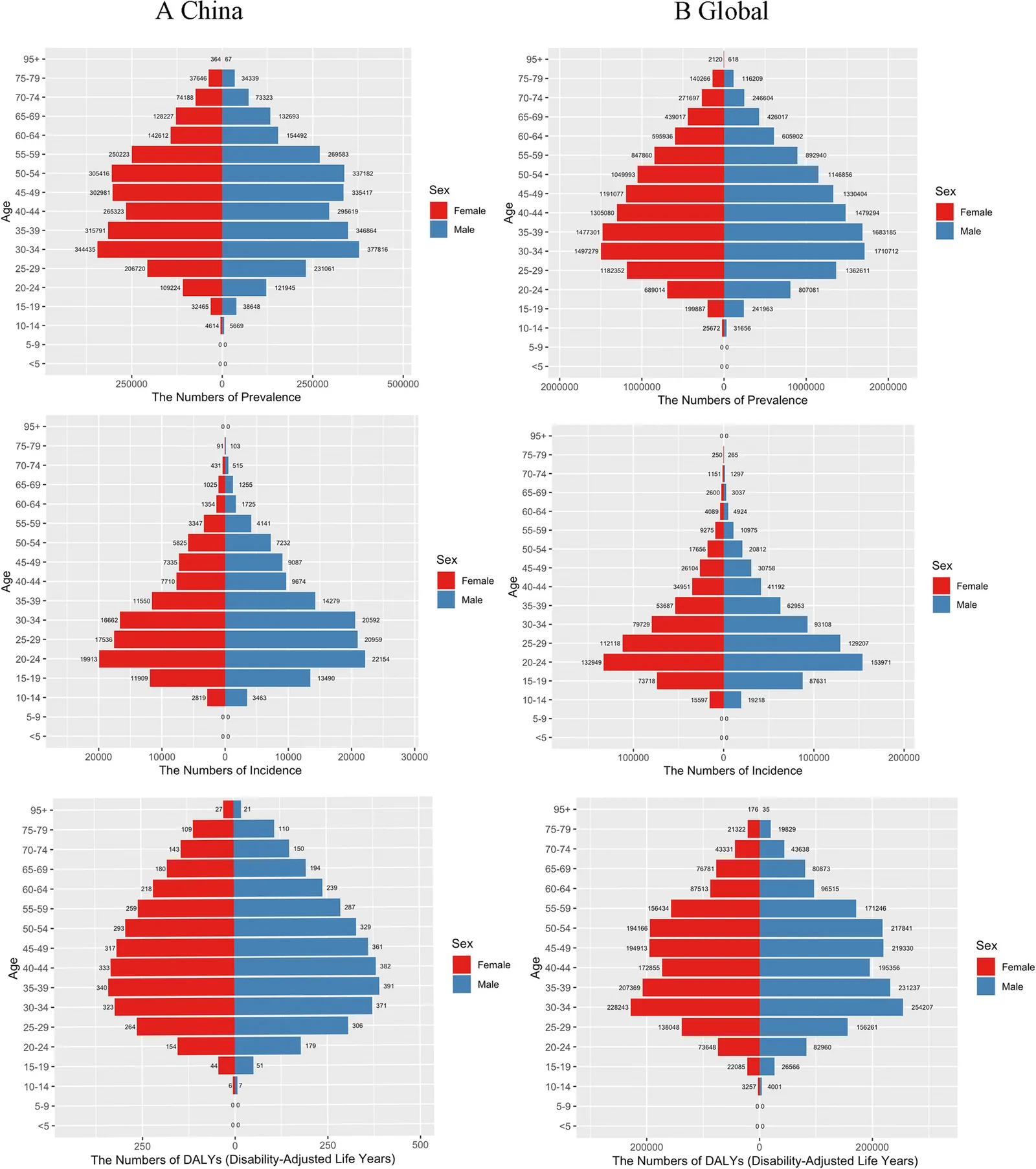
Two-sided plots of the burden of schizophrenia by age and sex

The global prevalence of schizophrenia peaked at the age of 30–34 years, with 1,710,712 cases in males and 1,497,279 cases in females. The global incidence rates for males and females peaked at 20–24 years, with 153,971 cases in males and 132,949 cases in females. The global DALYs also peaked at 30–34 years, with 254,207 cases in males and 228,243 cases in females, with males showing a higher burden than females (Figs. 2 and 3).

The China prevalence of schizophrenia peaked at the age of 30–34 years, with 377,816 cases in males and 344,435 cases in females. The China incidence rates for males and females peaked at 20–24 years, with 22,154cases in males and 19,913 cases in females. The China DALYs also peaked at 30–34 years, with 391 cases in males and 340 cases in females, with males showing a higher burden than females (Figs. 2 and 3).

### The time trend of the burden of schizophrenia by gender from 1990 to 2021

**Fig. 4:**
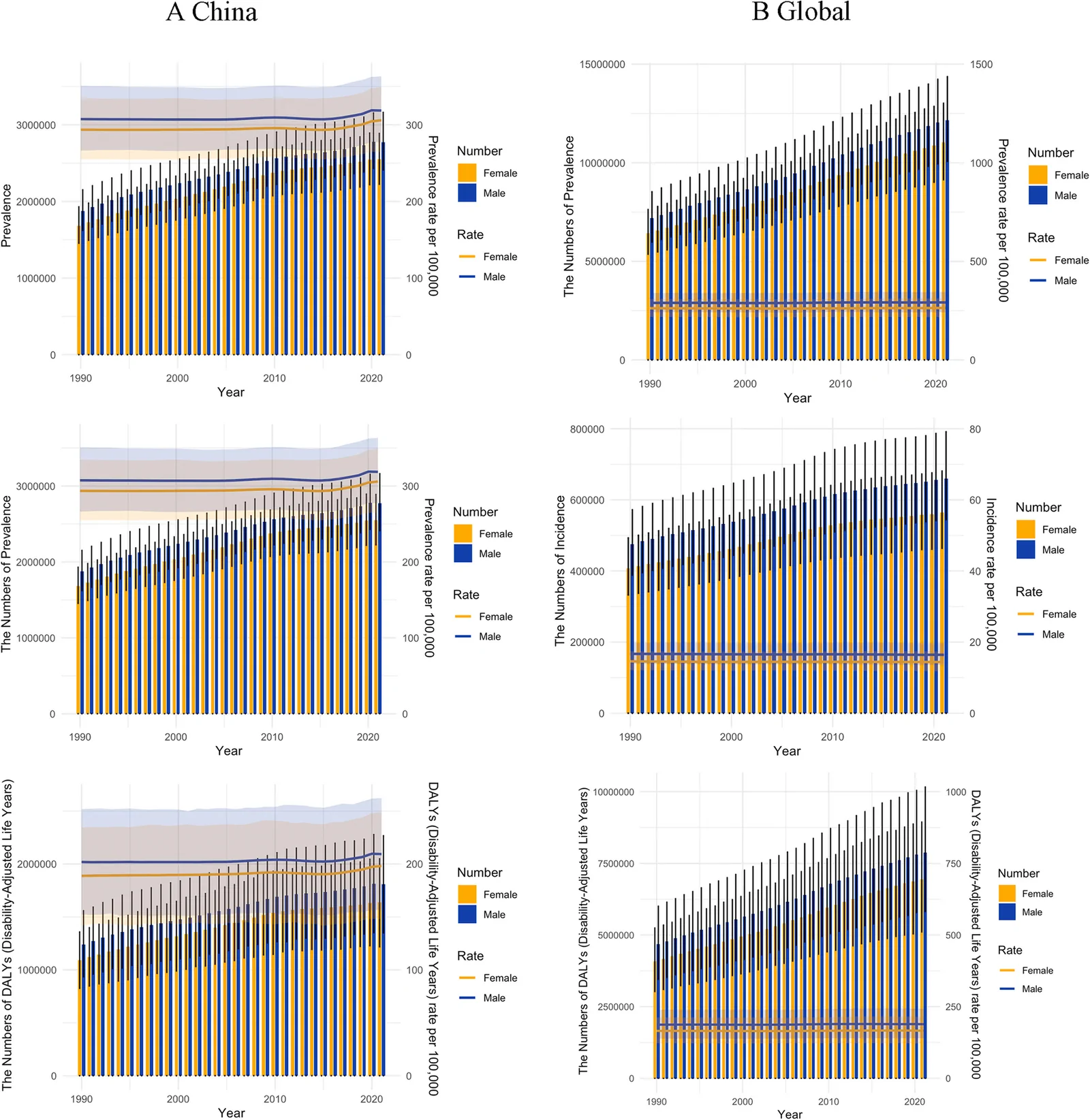
Biaxial plot of time trends in the burden of schizophrenia by gender

From 1990 to 2021, the number of cases of schizophrenia, including prevalence, incidence, and DALYs, has continued to increase in both China and globally, with the number of males being higher than females. However, there was no significant change in the age-adjusted prevalence, incidence, and DALYs (Fig. 4).

### The forecast of the burden of schizophrenia from 2022 to 2030

**Fig. 5:**
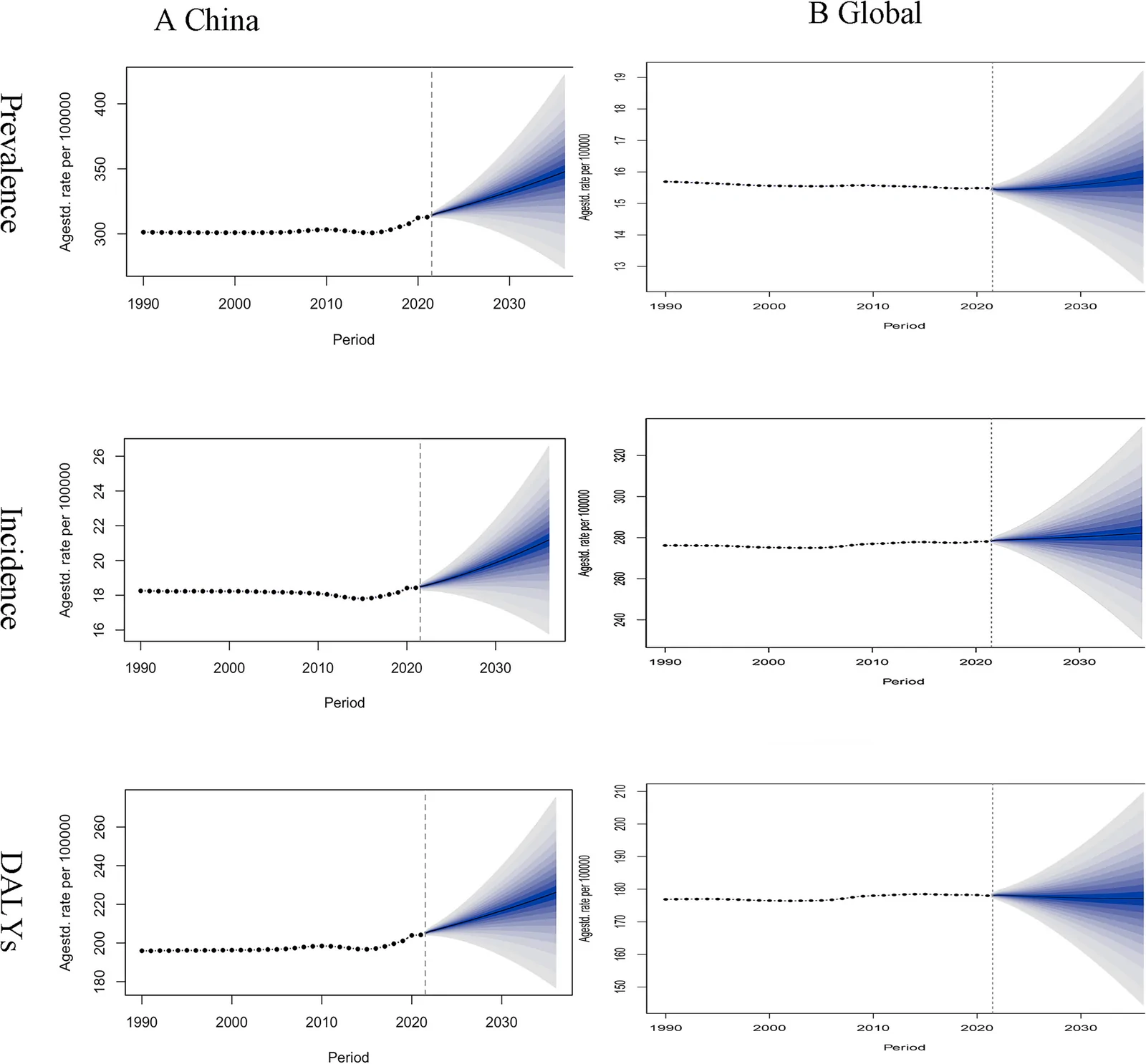
Plot of the predicted burden of schizophrenia

Based on the BAPC model, the forecast shows an increasing trend in the global and Chinese prevalence, incidence, and DALYs of schizophrenia from 2022 to 2030. By 2030, the global age-standardized prevalence, incidence, and DALYs of schizophrenia are projected to reach 280.36, 15.59, and 177.31 per 100,000 people, respectively. In China, the age-standardized prevalence, incidence, and DALYs are expected to reach 332.58, 19.87, and 216.67 per 100,000 people, respectively (Fig. 5 and Table 2).

**Table 2:** Projected burden of disease globally and in China 2022–2030

Years	Global			China		
	Age-standardized prevalence rate	Age-standardized incidence rate	Age-standardized DALYs rate	Age-standardized prevalence rate	Age-standardized incidence rate	Age-standardized DALYs rate
2022	278.73	15.44	178.19	315.62	18.55	205.98
2023	278.92	15.45	178.07	317.57	18.68	207.21
2024	279.11	15.45	177.95	319.56	18.83	208.47
2025	279.29	15.47	177.82	321.61	18.98	209.76
2026	279.48	15.48	177.70	323.69	19.14	211.07
2027	279.70	15.50	177.59	325.84	19.31	212.42
2028	279.92	15.53	177.50	328.03	19.49	213.81
2029	280.14	15.56	177.40	330.27	19.68	215.22
2030	280.36	15.59	177.31	332.58	19.87	216.67

## Discussion

To the best of our knowledge, this is the first analysis using the GBD 2021 database. Although there is some overlap between the present study and that of Solmi et al [32] in terms of study topics and data sources, the two studies were based on different versions of the GBD data. Solmi et al. used the GBD 2019 data, whereas the present study used the updated GBD 2021 data. As a result, there are some differences in the estimates of 1990 global age-standardized prevalence, incidence, and disability-adjusted life years (DALYs). For example, our estimates were 275.78, 15.64, and 176.61, respectively, compared with 289.92, 16.87, and 185.19, respectively, reported by Solmi et al. The main reason for the differences with the results of Solmi et al. (2023) is the difference in the version of data used and the way it was processed.Solmi et al. were based on GBD 2019 data, while this study uses the updated GBD 2021 data.Each update of the GBD database incorporates new data sources, optimizes the modeling methodology, and adjusts for the relevant covariates, resulting in the possibility that the estimates may be slightly different even for the same year (e.g., 1990).The results of Solmi et al. (2023) are not the same as the results of the GBD database. estimates may be slightly different even for the same year (e.g., 1990). In addition, we extracted standardized, globally aggregated data directly through the GBD results tool to ensure consistency of data sources and reproducibility of methods, whereas Solmi et al. may differ in country or population selection. Finally, this study includes the most recent data up to 2021, which is a more comprehensive reflection of trends in recent years than the Solmi et al. study that only goes up to 2019, and although this has limited impact on the 1990-specific estimates, it may cause some differences in directional judgments in the trend analysis.

Our research utilizes the latest data and specifically focuses on data from China. The study estimates that the global prevalence of schizophrenia is approximately 13.6 million cases, with an age-standardized rate of 275.78 per 100,000. The global incidence is about 1.20 million cases, with a DALY of 15.43 per 100,000; the global DALY is 14.8 million, with a rate of 177.75 per 100,000. In China, the prevalence is 5.30 million cases (300.81 per 100,000), the incidence is 236,175 cases (18.36 per 100,000), and the DALY is 3.40 million (203.88 per 100,000). From 1990 to 2021, both the standardized prevalence rate and DALY rate in China have shown an upward trend, with an EAPC of 0.12 and 0.04, respectively. The global age-standardized incidence rate has decreased, with an EAPC of −0.04. According to the BAPC model, predictions indicate that from 2020 to 2030, the prevalence, incidence, and DALY rates of schizophrenia will continue to rise globally and in China.

From 1990 to 2021, the global prevalence, incidence, and Disability-Adjusted Life Years (DALYs) of schizophrenia have all shown an upward trend. Although the global age-standardized prevalence and DALY rates have a relatively slow annual change rate (EAPC) of 0.02%, this still signals the continued accumulation of the burden of schizophrenia. The decline in global incidence (EAPC of −0.04%) may be related to improvements in early diagnosis and treatment, especially the gradual improvement of healthcare services in some low-income countries and regions [33]. However, this downward trend requires more evidence to support it, such as the promotion of more efficient preventive measures or interventions [34]. It is noteworthy that Qatar shows the greatest relative change in prevalence, incidence, and DALYs, a phenomenon that may be related to the country''s unique social and healthcare system structure [35]. As a high-income country, Qatar''s higher schizophrenia diagnosis rate may reflect the success of its public health system in identifying and reporting mental illnesses [36]. On the other hand, the rapid urbanization process, social pressure, and high immigrant population may have exacerbated the mental health burden in the region. Therefore, Qatar’s case not only reflects the real increase in disease burden but may also be a “byproduct” of improvements in healthcare services [37].

Particularly from 1990 to 2021, the chine growth rates of age-standardized prevalence (EAPC of 0.12%) and DALY rates (EAPC of 0.14%) maybe both higher than the global levels. The rising burden of schizophrenia in China is mainly attributed to a combination of factors, including population aging, accelerated urbanization, increased diagnosis rates due to improved accessibility of mental health services, and a general rise in psychosocial stress [38, 39]. However, since we did not make statistical inferences, these claims might have been exaggerated. Therefore, we still need to be cautious about these results.

In China, the diagnosis and treatment of schizophrenia still show significant regional disparities. Although progress has been made in mental health in recent years, particularly with the improvement of healthcare services in large cities, mental health services in remote areas remain scarce, and the social stigma surrounding mental health continues to be a major obstacle [40]. Additionally, with the aging population, the overall burden of mental illness is expected to increase further. During China''s rapid economic growth and urbanization, the pressures brought about by social transformation may be a key driving force behind the rising schizophrenia burden. Employment pressure, increasing living costs, and inadequate social security are all factors that may exacerbate mental health issues [41]. Among the younger population, mental health problems are particularly prominent, which may be closely related to excessive competition, academic pressure, and job insecurity [42].

Globally, the burden of schizophrenia is generally heavier among men. According to the findings, the highest burden of schizophrenia in both global and Chinese populations occur in the 30–34 age group, with the male population bearing a significantly heavier burden than females [43]. This phenomenon aligns with the known gender differences in schizophrenia, where men typically exhibit more pronounced early-onset characteristics and more severe symptoms. The pathogenesis of schizophrenia may be related to factors such as higher biological susceptibility in men, genetic factors, and societal pressures related to gender roles [44]. Although men dominate the burden of schizophrenia, the burden among women should not be overlooked. In China, the increasing trend in schizophrenia burden among women suggests that female populations are facing greater mental health risks, which may be related to social environment, role expectations, and factors such as childbirth [45]. Therefore, when formulating intervention policies, gender differences must be considered, especially the unique risk factors faced by women in different social environments. The age distribution of schizophrenia shows similar trends globally and in China, with the highest prevalence and DALYs occurring in the 30–34 age group, and the highest incidence occurring in the 20–24 age group. This indicates a clear trend towards younger age onset of schizophrenia. Early-onset, early diagnosis, and early intervention will be key in reducing this burden [46]. The early onset of schizophrenia may be intertwined with genetic susceptibility, environmental stressors, and neurodevelopmental issues during adolescence. Therefore, mental health interventions targeting younger populations are especially important. Early mental health education, screening, and interventions for adolescents and young adults may significantly reduce the long-term burden of the disease [47].

Based on the BAPC model, global and Chinese schizophrenia burdens are expected to continue increasing. By 2030, the age-standardized prevalence, incidence, and DALYs for schizophrenia are projected to further rise. The predicted values for global schizophrenia prevalence, incidence, and DALYs are 280.36, 15.59, and 177.31 per 100,000 respectively, while the corresponding predicted values for China are 332.58, 19.87, and 216.67 per 100,000. This prediction highlights the long-term increasing trend of schizophrenia burden and raises higher demands for public health systems [48, 49]. As the global population ages and mental health issues gain more attention, the burden of schizophrenia is expected to continue to grow. Especially in low- and middle-income countries, where population growth, urbanization, and insufficient healthcare resources prevail, the challenges may become even more severe [50]. Therefore, it is urgent to formulate and implement sustainable mental health policies and enhance the accessibility and quality of mental health services.

### Strengths and limitations

This study has multiple advantages. First, the data were obtained from the global authoritative GBD 2021 database, which covers several indicators on the incidence, prevalence, and DALYs of schizophrenia between 1990 and 2021, making the data comprehensive, reliable, and highly representative and comparable. Secondly, the study adopted the ASR for comparison, which avoids the bias caused by the difference in population age structure and makes the data between China and the world more scientific in the side-by-side comparison. In addition, the trend of disease burden from 1990 to 2021 was analyzed through the Joinpoint regression model, which was able to accurately capture the inflection points and development patterns, making the analysis more informative. As for prediction, the study utilized the BAPC model, combined with the INLA and MCMC methods, to scientifically predict the burden of schizophrenia from 2022 to 2030, which provides a prospective reference for future public health decision-making.

Nevertheless, this study has some limitations. First, the prediction model was extrapolated based on historical trends only and did not consider potential future changes in risk factors or improvements in prevention and treatment measures, which may affect the accuracy of the prediction. Second, as there is no evidence that schizophrenia can directly cause death, the GBD database does not provide relevant mortality data to assess the year of life lost (YLL) due to premature death, which may lead to an underestimation of the disease burden. In addition, the scarcity of basic data in some areas requires estimation through modeling, and although Bayesian methods have been used to deal with uncertainty, the models themselves may still be biased. Finally, this study failed to further disaggregate the different subtypes or severity of schizophrenia, limiting in-depth understanding of disease heterogeneity, and did not incorporate social, economic, and other related burdens, which may affect the comprehensiveness of the results.

## Conclusion

The global burden of schizophrenia is increasing, particularly in China, where the trend is especially pronounced. In response to this trend, Firstly, the mental health service system should be further strengthened, especially targeted interventions in populations with higher prevalence and burden of DALYs, such as strengthening early screening and mental health education in the high prevalence age group of young adults. Second, the results suggest that male populations bear a higher burden of disease, and therefore efforts to identify and intervene in men''s mental health problems should be strengthened, including the promotion of gender-sensitive diagnostic and treatment pathways and rehabilitation service design. In addition, as the burden will continue to rise in the future, there is a need to increase the coverage of comprehensive prevention and control measures, such as early intervention, comprehensive treatment, and community rehabilitation for schizophrenia, through rational allocation of resources and long-term strategic planning. Finally, policy makers should make full use of the burden prediction data provided in this study and incorporate them into national and local mental health service planning, with particular attention to service accessibility and equity for high-risk populations, to minimize the long-term impact of schizophrenia on society and families.

## Supplementary Information

Supplementary Material 1.

## Data Availability

The data used for the analyses are available upon reasonable request from the first author. The data sets supporting the conclusions of this article are included within the article and its additional files.

## Abbreviations

DALYs: Disability-adjusted life years
BAPC: Bayesian age-period-cohort
YLDs: Years Lived with Disability
ASRs: Age-standardized rates
CI: Confidence intervals
YLL: Years of Life Lost
AAPC: Average Annual Percent Change
MCMC: Markov Chain Monte Carlo
DIC: Deviance Information Criterion
