# Supplementary material for: Global burden and prediction study of schizophrenia 1990–2030: comparison with China
Source: BMC Psychiatry. 2025 Oct 9;25:955. doi: 10.1186/s12888-025-07168-6 (PMC12512590; doi:10.1186/s12888-025-07168-6)
Supplement: Supplementary file 1 — Supplementary Material 1. [file 12888_2025_7168_MOESM1_ESM.docx]

Table S1 Changes in the burden of schizophrenia worldwide from 1990 to 2021

| Incidence | | | | DALYs | | | | Prevalence | | | |
| --- | --- | --- | --- | --- | --- | --- | --- | --- | --- | --- | --- |
| location | case_1990 | case_2021 | change | location | case_1990 | case_2021 | change | location | case_1990 | case_2021 | change |
| Qatar | 8.74 | 61.45 | 6.03 | Qatar | 92.17 | 766.53 | 7.32 | Qatar | 141.37 | 1181.40 | 7.36 |
| Equatorial Guinea | 5.20 | 23.90 | 3.59 | United Arab Emirates | 369.00 | 2540.08 | 5.88 | United Arab Emirates | 565.14 | 3941.27 | 5.97 |
| United Arab Emirates | 35.73 | 148.34 | 3.15 | Maldives | 29.35 | 135.24 | 3.61 | Jordan | 669.03 | 3067.99 | 3.59 |
| Afghanistan | 113.91 | 425.11 | 2.73 | Jordan | 433.78 | 1971.46 | 3.54 | Maldives | 45.12 | 206.27 | 3.57 |
| Jordan | 57.37 | 200.40 | 2.49 | Equatorial Guinea | 40.13 | 181.60 | 3.52 | Equatorial Guinea | 63.99 | 284.40 | 3.44 |
| Cameroon | 138.07 | 458.35 | 2.32 | Gulf Cooperation Council | 3266.03 | 13573.90 | 3.16 | Gulf Cooperation Council | 5034.46 | 21106.17 | 3.19 |
| Djibouti | 6.12 | 19.47 | 2.18 | Djibouti | 45.77 | 178.52 | 2.90 | Djibouti | 70.51 | 275.99 | 2.91 |
| Angola | 134.46 | 422.23 | 2.14 | Saudi Arabia | 2125.70 | 7928.33 | 2.73 | Saudi Arabia | 3283.94 | 12353.06 | 2.76 |
| Niger | 100.37 | 312.10 | 2.11 | Bahrain | 89.44 | 327.01 | 2.66 | Bahrain | 137.62 | 508.45 | 2.69 |
